# Support needs of Australians bereaved during the COVID-19 pandemic: A cross-sectional survey study

**DOI:** 10.1371/journal.pone.0304025

**Published:** 2024-06-06

**Authors:** Serra E. Ivynian, Fiona Maccallum, Sungwon Chang, Lauren J. Breen, Jane L. Phillips, Meera Agar, Annmarie Hosie, Jennifer Tieman, Michelle DiGiacomo, Tim Luckett, Jennifer Philip, Ann Dadich, Christopher Grossman, Imelda Gilmore, Janeane Harlum, Irina Kinchin, Nicholas Glasgow, Elizabeth A. Lobb

**Affiliations:** 1 Faculty of Health, IMPACCT, University of Technology Sydney, Ultimo, NSW, Australia; 2 Faculty of Health and Behavioural Sciences, School of Psychology, University of Queensland, St Lucia, QLD, Australia; 3 Curtin School of Population Health and Curtin enAble Institute, Curtin University, Perth, Western Australia; 4 Faculty of Health, School of Nursing, Queensland University of Technology, Kelvin Grove, QLD, Australia; 5 School of Nursing & Midwifery, University of Notre Dame Australia, Darlinghurst, NSW, Australia; 6 St Vincent’s Health Network Sydney, Darlinghurst, NSW, Australia; 7 Faculty of Health Sciences, RePaDD, Flinders University, Adelaide, SA, Australia; 8 Department of Medicine, St Vincent’s Hospital, University of Melbourne, Fitzroy, VIC, Australia; 9 School of Business, Western Sydney University, Parramatta, NSW, Australia; 10 Bethlehem, Caulfield South, VIC, Australia; 11 District Palliative Care Service, Liverpool Hospital, Liverpool, NSW, Australia; 12 Centre for Health Policy and Management, School of Medicine, Trinity College Dublin, the University of Dublin, Dublin 2, Ireland; 13 Medical School, Australian National University, Canberra, ACT, Australia; 14 Department of Palliative Care, Calvary Health Care Kogarah, Kogarah, NSW, Australia; Rey Juan Carlos University: Universidad Rey Juan Carlos, SPAIN

## Abstract

**Background:**

COVID-19 disrupted access to bereavement support. The objective of this study was to identify the bereavement supports used by Australians during the COVID-19 pandemic, perceived helpfulness of supports used, prevalence and areas of unmet support need, and characteristics of those with unmet support needs.

**Methods:**

A convenience sample of bereaved adults completed an online questionnaire (April 2021-April 2022) about their bereavement experiences including support use and perceived helpfulness, unmet support needs and mental health. Multiple logistic regression was conducted to determine sociodemographic correlates of unmet needs. Open-ended responses were examined using content analysis to determine key themes.

**Results:**

1,878 bereaved Australians completed the questionnaire. Participants were mostly women (94.9%) living in major cities (68%) and reported the death of a parent (45%), with an average age of 55.1 years (SD = 12.2). The five most used supports were family and friends, self-help resources, general practitioners, psychologists, and internet/online community groups. Notably, each was nominated as most helpful and most unhelpful by participants. Two-thirds (66%) reported specific unmet support needs. Those with unmet needs scored lower on mental health measures. Correlates of unmet needs included being of younger age, being a spouse or parent to the deceased; reporting more impacts from public health measures, and not reporting family and friends as supports. The most frequent unmet need was for social support after the death and during lockdown.

**Conclusions:**

This study demonstrates the complexity of bereavement support needs during a pandemic. Specialised grief therapy needs to be more readily available to the minority of grievers who would benefit from it. A clear recommendation for a bereavement support action plan is to bolster the ability of social networks to provide support in times of loss. The fostering of social support in the wake of bereavement is a major gap that needs to be addressed in practice, policy, and research.

## Introduction

Evidence suggests that most people will find ways to adapt to bereavement and that few will need specialist grief therapies [1–3]. Concurrently, however, bereaved people have reported difficulties accessing support, be that informally from family and friends, or formally from psychologists and grief counsellors [2,4–7]. Access difficulties were likely compounded by the COVID-19 pandemic due to travel restrictions and limited face-to-face interactions with family, friends, and formal health services. This led to calls from grief researchers to prepare for a ‘silent epidemic of grief’[8,9]. Internationally several studies have found that pandemic-related challenges have impacted on the support needs of the bereaved and the support sources available [10–13]. The support needs of bereaved people in Australia during the pandemic have not been examined.

Experiences in Australia during 2020 and 2021 differed markedly from the global situation, with considerably fewer infections and deaths from COVID-19. At the same time, however, Australia experienced some of the longest and most restrictive public health measures in Western countries, introduced to reduce the spread of COVID-19 and protect health workers and the community [14]. At various times, gatherings with family and friends who did not live in the same household were heavily restricted or prohibited, and many formal supports closed or shifted online, including mental health services. International borders closed in March 2020 for almost two years, citizens had to seek permission to leave or re-enter the country and state borders closed throughout 2020–2021 inhibiting travel within the country [15]. Demand for mental health services grew. In January and February 2022, psychologists reported a marked increase in wait times, worked an average of 17 additional hours unpaid a week to accommodate higher demand, and one-third closed their books to new clients [16]. Together, these factors suggest that many bereaved Australians might have been left with unmet support needs.

In the context of providing bereavement support, it is increasingly recognised that a public health approach is needed. This approach recognises that people may benefit from different types and sources of support depending on their risk of developing complex grief reactions. It involves tailoring support approaches to meet bereaved people’s needs at universal, selective, and indicated levels [17–19]. At a universal level, there is the provision of basic information about grief and compassion to all bereaved people, but with most support coming from existing social networks. Social support is modifiable and can helpfully influence the grieving process, making it an effective way to reduce the risk of deleterious grief outcomes [20,21]. When the recipient perceives it as helpful, social support is one of the strongest determinants of positive psychosocial outcomes after bereavement [22–24]. At a selective level, additional community support is suggested to reduce the likelihood of further complications. This could involve peer support from grief support groups and organisations or support from a general practitioner. At an indicated level, which refers to people showing signs of complicated reaction or high levels of mental health distress, it is recommended that supports involve targeted therapies delivered by relevant experts [17,25,26]. For example, prolonged grief disorder, a condition which affects about 10% of bereaved individuals and is associated with significant negative health and mental health impacts, responds selectively to psychotherapies designed specifically to address it [27–29]. Prior to the pandemic, one-third of bereaved Australians reported not receiving the support they would have liked [4]. Additionally, support when provided is not always perceived by the recipient as helpful or timely [2].

The National COVID-19 Bereavement Project was established to quantify the support needs and mental health effects of Australians bereaved from any cause during the COVID-19 global pandemic. Over 2000 Australians bereaved from any cause during the first two years of the pandemic completed the survey. Maccallum et al [30] reporting on the mental health outcomes, found that 53% of sample experienced elevated symptoms of grief and/or depression and anxiety. Almost 20% of the sample reported concurrent high levels of all three. Importantly, the experience of social isolation and loneliness was an independent correlate of high symptoms, relative to low symptoms.

In this article, we present primary findings from the National COVID-19 Bereavement Project related to bereavement support use. Our aims were to 1) identify the bereavement supports used by participants, 2) examine the perceived helpfulness of the supports used, 3) establish the prevalence of unmet needs, 4) determine the sociodemographic correlates and mental health measures associated with having unmet support needs, and 5) identify the nature of the unmet support needs. This information will enable identification of vulnerable groups and development of strategies to address unmet needs of bereaved Australians now, and in future pandemics.

## Methods

### Study design

Presented here is a cross-sectional survey study which formed part of a larger mixed-method longitudinal study called the National COVID-19 Bereavement Project.

### Participants and procedures

A convenience sample of bereaved people completed a twenty-minute online questionnaire indexing end-of-life and bereavement experiences. Inclusion criteria were: Australian adults aged 18 years or over who experienced a death (from any cause) of a relative or friend between January 2020 and February 2022 (inclusive); self-identified as a carer, family member, or close friend of the decedent; at least two months post-bereavement; and had adequate English comprehension to complete the questionnaire. There were no additional exclusion criteria. The questionnaire was administered online via REDCap hosted by (blinded for review). The study was advertised via social media as well as through national community and consumer organisations that distributed questionnaire information through their networks from April 2021 to April 2022 (inclusive). Most participants were recruited during two waves of Facebook and Instagram advertising (September 15 to October 15, 2021, inclusive and February 15 to March 15, 2022, inclusive). Potential participants accessed the participant information sheet detailing the study purpose, time commitment, where data would be stored, and how data would be used to protect confidentiality. All volunteers provided informed consent prior to participation by selecting ‘I have read the information above and agree to taking part in this survey’. Volunteers could not access the survey questions if they did not click consent. The study protocol was approved by the (blinded for review) Human Research & Ethics Committee. Ref (blinded for review). No incentives were offered. Participants could complete the survey without answering all questions, but cases were considered valid for this analysis if the assessment of unmet support needs question was answered.

### Questionnaire development and content

The questionnaire was designed and pilot-tested by the researchers with consumer input (blinded for review). The questionnaire was presented in five sections. Section one included questions about the decedent and the death. Section two pertained to end-of-life experiences, including health care use, interactions with clinicians, and the impact of COVID-19 public health restrictions (see S1 File). Section three, the focus of this article, pertained to supports accessed after the decedent’s death, the (un)helpfulness of these supports, and whether people had unmet support needs. Section four focused on emotional, functional, and social outcomes, and Section five indexed participant sociodemographic characteristics, including employment and living arrangements.

#### Assessment of use and helpfulness of bereavement supports

Participants were provided with a list of 17 informal, community, and formal supports adapted from previous research [1,13] and were asked to indicate those they had accessed following bereavement. Participants were then asked to select the support they perceived as most helpful. Following this, they were given the opportunity to respond to two open-ended questions. The first asked them to describe the reasons they selected a particular support as most helpful. The second asked them if any of the supports were unhelpful and in what way.

#### Assessment of unmet support needs

In section 3 of the questionnaire, participants were also asked if, overall, they felt they: ‘*got as much support as I needed’* (i.e., needs met)*; ‘did not feel I needed support’* (i.e., needs met); *‘got some support but not as much as I needed’* (i.e., needs unmet)*; or ‘did not get the support that I needed’* (i.e., needs unmet). If this question was left unanswered, the case was excluded from analyses reported here. Support needs were categorised into two groups: ‘needs met’ or ‘needs unmet’. Participants could also describe specific unmet needs via an open-ended question. Participants were also asked to reflect on their experience and identify supports that might have helped that they did not or could not access. Options were in accordance with the tiers of the public health model of bereavement support and included: information (e.g., brochures, leaflets); community-based support (e.g., grief support groups, community groups, peer support); professional support (e.g., counsellor, psychologist); don’t know; no; and/or other. Participants could select more than one response.

#### Mental health measures

Depressive symptoms were assessed using the self-report nine-item Patient Health Questionnaire (PHQ-9) [31,32]. Participants responded to each on a four-point Likert scale, indicating the frequency of the symptom over the past two weeks (0 = not at all, 1 = several days, 2 = more than half the days, 3 = nearly every day). Scores of ten or above suggest at least moderate levels of depression.

Symptoms of anxiety were assessed using the self-report seven-item Generalised Anxiety Disorder scale (GAD-7) [33]. Participants responded to items reflecting the experience of anxiety symptoms over the last two weeks. Each item was scored on a four-point Likert scale (0 = not at all, 3 = nearly every day). Scores of ten or above suggest at least moderate levels of anxiety.

The Prolonged Grief Scale-Revised (PG-13-R) is a validated measure of prolonged grief symptoms including yearning, disbelief, emotional pain and numbness, meaninglessness, and loneliness [34] where participants responded to ten items indicating symptom severity (1 = not at all, 5 = overwhelmingly). Scores of thirty or above at 12 months, post-bereavement, suggest possible prolonged grief disorder.

### Data analysis

Data were analysed using SPSS V26 software. Univariate relationships between demographics and COVID-19 impacts and support needs were examined using chi-square for categorical variables and independent sample *t*-tests for continuous variables. Information about supports used are reported using descriptive statistics. Multiple logistic regression was used to determine multivariate relationships between unmet needs and sociodemographics, COVID-19 public health measures, and supports accessed. Variables significantly (*p*<0.05) associated with unmet needs on univariate analysis were entered into multivariate models. Final models were determined by considering collinearity and goodness-of-fit assessed using the Hosmer-Lemeshow test. No missing data were imputed. Open-ended responses were coded by three researchers (blinded for review) in NVivo software using conventional content analysis [35]. Inductive coding was undertaken on responses, and descriptive categories were developed by grouping similar codes. Categories were refined on two separate occasions through mapping all categories and discussing links, and parameters of each category. Disagreements were discussed among the three researchers until consensus was reached.

## Results

### Characteristics of the sample

A total of 1,878 bereaved people (94.9% women; mean age 55.1 years) completed the survey (see Table 1 and S2 File). Most resided in major cities (68.4%), were university educated (87.7%), employed (59.8%), and partnered (59.7%). One-quarter reported living alone (25.9%). Most participants reported they had some unmet support needs (62.0%). People with unmet needs were significantly younger and indicated higher levels of distress on the mental health measures. They also reported different kinship relationships of the deceased and younger age of the decedent. Cause of death did not differ between groups, nor did relationship status or living alone. People with unmet support needs reported higher levels of anxiety, depression, and prolonged grief symptoms (see Table 1). For space, the experience of COVID-19 restrictions for the total sample and differences between groups are reported in S3 File.

**Table 1:** Participant characteristics, death characteristics, and mental health measures by support needs.

	Total (*n* = 1,878)	Support needs met (*n* = 714)	Unmet support needs (*n* = 1,164)	*p*-value ^i^
	*n* (%)			
*Participant characteristics*				
Age (years)—mean (SD)	55.1 (12.2)	58.1 (11.6)	53.2 (12.3)	< .001
Gender ^a^				
Female	1648 (94.9)	627 (94.4)	1021 (95.2)	.63
Male	79 (4.6)	34 (5.1)	45 (4.2)	.63
Remoteness ^b^				.99
Major Australian cities	1016 (68.4)	383 (68.3)	633 (68.4)	
Inner regional Australia	344 (23.1)	131 (23.4)	213 (23)	
Outer regional/Remote/Very remote Australia	126 (8.5)	47 (8.4)	79 (8.5)	
Born in Australia ^c^	1085 (79.3)	411 (79.3)	674 (79.3)	.98
Highest level of education ^d^				.38
Year 12	183 (12.2)	66 (11.7)	117 (12.6)	
Undergraduate	487 (32.5)	174 (30.8)	313 (33.6)	
Postgraduate	827 (55.2)	325 (57.5)	502 (53.9)	
Employment ^e^				.74
Employed	897 (59.8)	342 (60.4)	555 (59.4)	
Not in workforce	554 (36.9)	208 (36.7)	346 (37)	
Looking for work	49 (3.3)	16 (2.8)	33 (3.5)	
Relationship status ^f^				.20
Married/Defacto	882 (59.7)	353 (63.1)	529 (57.6)	
Widowed	248 (16.8)	85 (15.2)	163 (17.8)	
Single	209 (14.2)	75 (13.4)	134 (14.6)	
Separated/Divorced	138 (9.3)	46 (8.2)	92 (10)	
Lives alone ^g^	387 (25.9)	142 (25.2)	245 (26.4)	.60
*Death characteristics*				
Relationship of the deceased				.007
Parent	850 (45.3)	314 (44.0)	536 (46.0)	
Partner	323 (17.2)	112 (15.7)	211 (18.1)	
Sibling	176 (9.4)	68 (9.5)	108 (9.3)	
Child	135 (7.2)	41 (5.7)	94 (8.1)	
Other family	265 (14.1)	126 (17.6)	139 (11.9)	
Other (not a family member)	129 (6.9)	53 (7.4)	76 (6.5)	
Age at death	70.1 (21.7)	73.90 (19.8)	67.75 (22.6)	< .001
Cause of death ^h^				.74
Cancer	559 (31.3)	218 (31.9)	341 (30.9)	
Chronic Health Condition	423 (23.7)	161 (23.6)	262 (23.8)	
Sudden health event or illness	414 (23.2)	153 (22.4)	261 (23.7)	
COVID-19 –related	51 (2.9)	16 (2.3)	35 (3.2)	
Injury/accident/suicide	185 (10.4)	78 (11.4)	107 (9.7)	
Other	154 (8.6)	57 (8.3)	97 (8.8)	
Died outside Australia	200 (10.6)	82 (11.5)	118 (10.1)	.76
Time since death (months)–mean SD	10.1 (5.9)	9.9 (5.8)	10.2 (6.0)	.28
*Mental health measures*				
Depression (PHQ-9)—mean (SD)	9.8 (7.0)	6.5 (5.7)	11.9 (7.0)	< .001
Anxiety (GAD-7)–mean (SD)	7.4 (6.0)	4.6 (4.7)	9.1 (6.1)	< .001
Prolonged grief (PG-13-R)—mean (SD)	27.4 (10.3)	22.1 (8.8)	30.7 (9.7)	< .001

^a^ Total n = 1736 for gender and n = 9 indicated ‘other’ or ‘prefer not to say’.

^b^ Remoteness classified using the using the Australian Statistical Geography Standard remoteness structure [36] which classifies Australia into five classes of remoteness based on a measure of relative access to services. Total n = 1486

^c^ Total n = 1368

^d^ Total n = 1497

^e^ Total n = 1500

^f^ Total n = 1477

^g^ Total n = 1494

^h^ Total n = 1786

^i^ *p* values reflect difference for each subgroup rather than overall totals.

### Use of bereavement supports

On average, participants used a mean of 2.3 (SD = 1.8) supports (see Table 2). Overall, family and friends (85.1%) and self-help resources (27.2%) (e.g., books, websites) were used most. One-fifth visited a general practitioner (21.5%) and 19.6% visited a psychologist. Approximately 17.6% accessed online support groups (e.g., via Facebook). Those with unmet needs sought support from more sources (see Table 2; mean = 2.5, SD = 1.9) than those whose support needs were met (mean = 2.1, SD = 1.8). Those with unmet support needs were less likely to report using family and friends for support (*p*<0.001) but more likely to report using self-help resources (*p*<0.001), internet/online community groups (*p*<0.001), informal bereavement support groups (*p* = 0.018), formal grief support groups (*p* = 0.034), general practitioners (p = 0.043), psychologists (*p* = 0.006), and psychiatrists (*p* = 0.008).

**Table 2:** Bereavement supports used, categorised by unmet support needs.

	Total (*n* = 1,878)	Support needs met (*n* = 714)	Unmet supports needs (*n* = 1,164)	*p*-value ^b^
	*n* (%)			
Number of supports used–mean (SD)	2.3 (1.8)	2.1 (1.7)	2.5 (1.9)	< .001
Supports used ^a^				
Family and friends	1598 (85.1)	641 (89.8)	957 (82.2)	<0.001
Self-help resources	510 (27.2)	156 (21.8)	354 (30.4)	<0.001
General practitioner	404 (21.5)	136 (19)	268 (23)	.043
Psychologist	368 (19.6)	117 (16.4)	251 (21.6)	.006
Online community support groups (e.g., Facebook)	331 (17.6)	83 (11.6)	248 (21.3)	<0.001
Grief counselling	204 (10.9)	66 (9.2)	138 (11.9)	.079
Religious leaders/organisations	158 (8.4)	69 (9.7)	89 (7.6)	.145
Legal professionals	139 (7.4)	57 (8)	82 (7)	.468
Financial professionals	68 (3.6)	22 (3.1)	46 (4)	.374
Advice or support line (e.g. Beyond Blue, Lifeline)	65 (3.5)	18 (2.5)	47 (4)	.091
Informal support groups for bereaved people	57 (3)	13 (1.8)	44 (3.8)	.018
Psychiatrist	57 (3)	12 (1.7)	45 (3.9)	.008
Social worker	51 (2.7)	20 (2.8)	31 (2.7)	.884
Palliative care service	50 (2.7)	20 (2.8)	30 (2.6)	.770
Grief support groups	41 (2.2)	9 (1.3)	32 (2.7)	.034
Local community organisations (e.g., community classes)	21 (1.1)	11 (1.5)	10 (0.9)	.182
Community services (e.g., meals on wheels)	18 (1)	8 (1.1)	10 (0.9)	.629
Other	80 (4.3)	28 (3.9)	52 (4.5)	.638

^a^ Participants could select more than one support.

^b^ *p* values reflect difference for each subgroup rather than overall totals.

### Perceived helpfulness of bereavement supports

Most participants indicated that family and friends were the most helpful support (66.7%), followed by psychologists (8.9%), self-help resources (4.9%), and grief counselling (4.9%) (see S4 File). In the open-ended question, the largest proportion of participants (27.9%) reported that nothing was unhelpful for them. The next largest proportion of participants indicated family and friends (23.9%) were unhelpful, followed by psychologists (4.7%). Open-ended responses presented in Table 3 provided some insight into why family and friends, community, and formal supports were perceived either as helpful or unhelpful.

**Table 3:** Reasons why supports were perceived as helpful or unhelpful as reported by participants in open-ended responses.

Support type	Reasons	Supporting quotes
Family and friends	Helpful • Shared experience • Pre-existing relationships—trust • Most readily available support • Emotional and practical support Unhelpful • Lack of face-to-face contact • Escalating family tensions compounded by COVID-19 • Platitudes/unhelpful comments • Stretched emotional capacity due to COVID-19 • Lack of sustained offers for support • Pressure to move on with life	*‘My children and their families are the reason I keep going each day*. *Without my family*, *including my 2 siblings and dear friends I would be lost*.*’—*Participant 1,178 *‘Well-meaning people who say stupid shit like- “Oh it’s a blessing—no more suffering” “How old was she"—that question made me want to punch them in the face*!*’—*Participant 2,468 *‘My family were too emotional to provide support to each other at the time’*–Participant 2,325
Community supports	Helpful • Readily available • Comfort in hearing/reading others’ experiences • Interacting with others with shared experience • Purpose and hope from shared beliefs Unhelpful • Non-specific • Detrimental to mental health—distressing reading/hearing others’ experiences • Lack of face-to-face availability	*‘At the time of my son’s death I was already reading a book that dealt with grief and how people dealt with it*. *It gave me hope that if other people could get through it I could*. *I found reading about people’s experiences from a number of sources quite helpful*.*’-* Participant 3,107 *‘Also watching other people grieve in a Facebook group has been hard*, *because they seem lost in that grief for many years*, *debilitated by it and that’s horrifying to think that could be me*, *and difficult because I can’t do anything to help them’*.*—*Participant 588
Formal health supports	Helpful • Specialist care • Targeted strategies for managing emotions • Objectivity–non-biased advice • Pre-existing relationships–trust • Referral to other mental health supports Unhelpful • Not specialised in /grief counselling • Pathologising grief rather than seeing it as a natural process • Cancelling sessions • Out-of-pocket cost • Delay in appointments (up to 15 months) • Telehealth unsuitable	*‘I felt I could talk openly and cry when I felt like it*. *There was no need to put on a brave face and pretend I didn’t hurt…Talking to someone who had no preconceived ideas about me*, *my Mum*, *the years of huge issues leading up to her death*, *was a blessing*.*’—*Participant 1,030 *‘Sometimes it feels like there needs to be a purpose with psychology*. *Without the purpose*, *the psychologist doesn’t know how to support you*. *Sometimes we just need to talk*. *Sometimes we just need to cry*. *We don’t always need to be ’fixed’*.*’—*Participant 4,017
Other—Government and lockdown	Unhelpful • Lack of vaccine availability • Harsh restrictions • Inconsistent government approach across Australian states • Government lack of pandemic planning regarding mental health support	*‘I was granted an exit permit after 7 days (way too late as mum had died while waiting) but my (age) daughter was refused one*. *It took another 3 days of protesting and appealing with the help of local doctors*, *psychologists and politicians for my daughter to be allowed to travel with me*. *The government was unbelievable—apparently it was more important for my daughter to stay in Australia because she might miss school than to be with her mum while burying her grandmother—they were very close’—*Participant 669
Other—Legal and financial supports	Unhelpful • Communication issues • Delays in processing	*‘The so-called bereavement team from (Bank) were so unhelpful*. *I had to continually harass them*, *they lost documents*, *they didn’t contact me*. *I had to initiate all contacts and they were so hard to get hold of—literally hours on the phone’—*Participant 1,358

Note. Community supports included: Self-help resources; grief support groups; community support groups; online support groups (Facebook, Reddit); religious leaders and organisations. Formal health supports included: Psychologists; grief counsellors; GPs; psychiatrists; phone support lines. Legal and financial supports included: Banks; Centrelink; coroner’s office; lawyers. Other supports such as government and legal and financial institutions were deemed as unhelpful only. Reasons for this are also presented in Table 3.

Reasons for family and friends being the most helpful included the shared experience of the death; the pre-existing nature of the relationship which facilitated feelings of support in their grief; being the most readily available support due to proximity or availability during lockdowns; and their ability to offer emotional and practical support in different ways. In contrast, family and friends were perceived as unhelpful due to lack of face-to-face availability, escalating family tensions compounded by COVID-19, platitudes, or other unhelpful comments; lack of sustained offers of support; the perception that emotional capacity of family and friends was already stretched due to COVID-19; and perceived pressure from friends and family to move on with life.

Community support groups and online supports, such as Facebook and Reddit were perceived as helpful as they allowed participants to interact with others who had similar experiences where participants felt they could express their emotions without judgement. In contrast, participants described community supports as unhelpful due to lack of specificity and increased distress from being exposed to others’ grief. Seeing/reading/hearing about others’ grief through community supports was perceived to worsen mental health. For similar reasons, social media was noted as unhelpful.

Formal supports were perceived to be helpful due to ability to provide specialist care, including targeted strategies for managing emotions; objectivity and ability to offer non-biased advice. Pre-existing relationships were once again noted, where trust had already been established. Participants felt that they could express emotions freely, where they did not feel comfortable doing so with family and friends. On the contrary, formal supports were perceived as unhelpful due to not being specialised in trauma/grief counselling, or the perception that the practitioner was trying to “fix” their grief, while the participants viewed grief as being a natural process. Other reasons included the out-of-pocket cost of formal supports, delay in appointments with some waiting up to 15 months, and others found telehealth unsuitable.

### Correlates of unmet support needs

Results of the multiple logistic regression analysis are summarised (see Table 4). Older age was associated with having needs met (OR = 0.96; 95%CI 0.95 to 0.96). Four COVID-19-related challenges were positively associated with unmet support needs; COVID-19 impacted their ability to care for the deceased (OR = 1.84; 95%CI 1.47 to 2.32); to know what was happening to them (OR = 1.58; 95%CI 1.16 to 2.16); to say goodbye (OR = 1.48; 95%CI 1.16 to 1.88); and, limited close contact with family and friends after the death (OR = 1.81; 95%CI 1.45 to 2.24).

**Table 4:** Associations of unmet needs with independent variables: Multiple logistic regression.

	Adjusted Odd Ratio (95% CI)
Older age of the respondent	0.96 (0.95,0.96)
Relationship to the deceased	
Partner	2.58 (1.56,4.28)
Child	1.95 (1.09,3.49)
Sibling	1.56 (0.91,2.69)
Parent	1.14 (0.73,1.77)
Other family	0.60 (0.36,0.99)
Other (not a family member)	Ref
COVID factors	
COVID-19 impacted my ability to care for them as I would have liked	1.84 (1.47,2.32)
My contact with close relatives or friends was limited	1.81 (1.45,2.24)
I was unaware of what was happening to them	1.58 (1.16,2.16)
I was unable to say goodbye as I would have liked	1.48 (1.16,1.88)
Support used	
Family and friends	0.47 (0.34,0.66)

### Categories of unmet needs

Participants responded to a multiple-choice question asking what supports they would have wanted but did not or could not access. In the order of increasing need, 11.6% indicated that information would have helped, 28.9% selected community-based supports, such as grief support groups, as well as community and peer support groups, and 29.3% selected professional supports. One-quarter were unsure what would have helped (25.6%) and 23.4% said nothing would have helped. A total of 1,233 participants (66%) provided an open-text response to a question inquiring about additional unmet needs (6% of these responses were excluded from analysis as they involved descriptions of grief reactions or the compounding difficulties they experienced during the pandemic, rather than specific unmet needs). Of the remaining responses, twelve categories of unmet needs were identified (see Table 5). The most common reported unmet need was for social support after the death. Participants described the need to give and receive in-person support from their family/friends living within and outside Australia; the need for family togetherness; physical comfort; and someone to ‘check in’ initially and periodically after the death. Linkage and access to professional mental health support during the pandemic was the second most reported unmet need. Participants also expressed a range of unmet needs in relation to the time of death. When the death occurred in an inpatient setting, many participants reported a need for compassion and flexibility in visiting rules before, during, and after death so they could be present at the time of death; say goodbye; and spend time with the body. The need for clear, timely and consistent communication from healthcare professionals and government was often noted, particularly during periods of public health restrictions where family/friends had limited or no access to inpatient settings. Participants reported the need for staff to inform family of prognosis and approaching death; prepare family for the dying process; answer questions; provide information about the cause of death; involvement in achieving last wishes; and ensure compassionate delivery of death notice to each family member. Clear provision of information and compassion from the government regarding public health restrictions was also reported, including the need for timely communication of changing restrictions; clear instructions on how to obtain travel exemptions; and clear communication regarding rules for restrictions, such as rules surrounding compassionate visits. Confusion over public health restrictions resulted in worry and concern.

**Table 5:** Categories of unmet needs as reported by participants to open-ended items.

Category	Mentions	Supporting quote
Need for social support after death and during lockdown	368	*“It was very hard and unusual to grieve in isolation… I’m used to when a family loses a loved one you all come together to grieve as a family*. *You cry together and you share stories of your loved one and laugh and remember good times*. *It was a strange experience not having that*. *It felt like we were "missing" something*.*”–*Participant 1,291
Need for professional mental health support	300	*“I felt helpless & alone*, *didn’t even know where to access support for such unusual situation*. *It’s so specific but there are thousands who have experienced this*, *Australia is a nation of people whose origins are oversees*, *(I am) wondering if specific grief training is needed for this*.*”–*Participant 2,117
Need for togetherness at the time of death and the chance to say goodbye	166	*“My children wished to see their father after he passed away (while he was still physically at the hospital before being transferred to a funeral home)*. *They had not been able to be present (when) he died due to restriction on visiting hours*, *visitor numbers and Covid state-wide curfew*. *This request was denied due to internal Covid restrictions*. *The hospital was in a rural area with no current Covid cases*, *clearly viewing a body that was not Covid positive was not going to endanger staff or my sons*. *This denial without logical reason was very distressing*.*”–Participant 876*
Need clear communication from health care professionals and government	165	*“I had called the* [state] *health hotline for clarification of compassionate grounds at one point but found them useless*. *They could not give a straight answer and said it was up to me to decide if it’s justified*. *At the time [politician] was constantly saying no visitors*, *there was never discussion to explain compassionate grounds clearly and it all felt too hard*, *so I just stayed by myself*.*”–*Participant 1,544
Need to commemorate	142	*“I think I needed to attend the funeral(s) in person*. *It felt like the whole situation was not real*. *I never left my home but found out a friend had passed away from [cause]*, *when only a week earlier a family member also died of [cause]*. *All this happened and I never left the house*. *I sat through 2 online funerals and never left the house or saw friends and family in person*. *It was surreal*.*”–*Participant 421
Need for practical support after death	107	*“I would have loved someone to come and help me with basic household cleaning and maintenance*. *And a meal from someone would have meant the world to me*.*”–*Participant 4,158
Need for carer support before and after death	96	*“I was bereaved and also caring for my brother who had moved in with us during lockdown before my [family member] suddenly and unexpectedly died*. *I could barely function*, *and I was responsible for looking after him*. *It was almost too much*. *I needed support*.*”–*Participant 414
Need for improved quality of care for the dying	54	*“Under staffing meant that carers were too busy to spend adequate time with each resident*.*”–*Participant 2,700
Need for support from workplaces	37	*“An employer who understood just because I wasn’t there when he died*, *I still need time to process and grieve”–*Participant 1,820 *“Work only provides 2 days bereavement leave*, *with no appreciation that I had just lost a close immediate family member and that I was not able to be present during her time in hospital or during death/funeral to support family members overseas*, *who were under extreme pressure and grief dealing with the death of their mother*, *and having to care for their father who was still in hospital with COVID*. *It was just business as usual after my 2-day leave and you are expected to function as if you haven’t just gone through and still going through something very traumatic*.*”–Participant 89*
Need for information and advice about grief	15	*“Advice for adjusting to life as a single parent and managing my own grief*.*”–*Participant 4,614
Need for increased access to palliative care	8	*“We used the [Name] Community palliative care when he left palliative care*, *but due to very limited staffing*, *they were not able to attend much until his last few days when there was a short visit once a day*.* *.* *. *If they had been able to attend more*, *the following may have been avoided*:*—Not having a full understanding of medications I was supposed to prepare and administer*.*—Not knowing how to help him toilet*, *wash*, *avoid bedsores*, *back issues*, *etc when he was bedbound*.*—Feeling isolated and alone*, *worrying there was more I could have been doing*, *but having no idea what*.*—Not being able to ever take a break or have more than a couple of hrs sleep at a time as we did not have a backup carer*, *or any access to respite care*.*”–*Participant 644
Need for government and public acknowledgement	3	*“Understanding and compassion from friends and the media and the community who spent all their time complaining about lockdown and public health measures when I had seen how important they were*. *I mean where is the memorial to Covid victims*? *Where is the public support*? *People are openly aggressive to me challenging how my family member died as they don’t want to believe Covid is real and it could affect them*. *We are the hidden victims*.*”–*Participant 549

## Discussion

This study found that Australians bereaved during the COVID-19 pandemic at all levels of need (universal, selective, and indicated) used multiple avenues of support during this time, that no support appeared universally helpful, and that those with unmet support needs were more likely to be younger; have experienced the loss of a partner or child; and were impacted by COVID-19 in ways that affected their ability to care for the dying, understand what was happening to them, be with friends and family, and say goodbye.

The most common and helpful support was indicated to be family and friends, which is consistent with pre-pandemic literature [2] and aligns with public health recommendations for the provision of universal bereavement support [17–19]. Importantly however, for many, friends and family were also described as the least helpful source of support. Some reasons for this were analogous to pre-pandemic reasons, such as limited empathy, insensitivity, and poor advice. However, other reasons for unhelpfulness were specific to the circumstances of public health measures, including limited face-to-face contact, the perception that others could not tolerate their grief during a global pandemic, and disrupted relationships due to opposing views on the pandemic, vaccination, and compliance with COVID-19 public health measures. These reasons could compound feelings of social disconnection where the bereaved are unwilling or unable to express their feelings in a social context due to the perceived negative consequences of doing so [23]. We also found evidence of this with participants reporting friends and family as too emotional themselves to be able to provide support, and thus did not seek their support or perceived them as unhelpful (see unhelpful supports table–see Table 3). Combined, these factors may contribute to people masking their grief [8] contributing to increased loneliness and social isolation, which has been linked with increased psychological distress in this sample [30] and others [22,23]. The Irish Hospice Foundation and the European Grief Model have recently proposed an additional tier to the public health model, where the three above are underpinned by grief literacy for all [18,19]. Grief literacy serves as a way to improve social support by helping people, communities and society to identify grief more readily and have the knowledge and skills to seek out relevant information and supports to avoid negative outcomes associated with grief [37,38] In a grief literate society, people would openly speak about their losses and be comfortable hearing of others’ rather than showing discomfort and masking grief [37].

It is important to recognised that despite the literature highlighting the potential value of social support for grieving persons, a systematic review on social support following bereavement concluded that the body of research is highly fragmented, with methodological flaws and omissions (i.e., student samples as proxies for the general community, numerous biases, limited controls, poor quality) [39]. Thus, calls to transfer grief support to social networks and communities unprepared to provide it must be avoided due to its potential to cause further harm [37]. These challenges identified here with social support are important to consider in pandemic planning for bereavement support, as these complicating factors might hinder use and perceived helpfulness, which are typically considered sufficient buffers against the deleterious effects of bereavement [24].

Perhaps it is not surprising that self-help resources were the second most used source of support given the limited availability of face-to-face social supports. Participants described the helpfulness of self-help resources in terms of its availability (e.g., book, online resources) and the comfort they drew from being able to connect with and read about others’ experiences. This might have facilitated the sense of belonging to a group with shared experiences and concerns. Pre-pandemic, a sense of belonging and shared experiences was a top reason why people felt family and friends were helpful [2]; however, during the pandemic, it appears self-help resources such as books and information on grief-related websites may have been able to address some of these needs. Self-help resources such as these reflect bereavement support recommended at the universal and selected level.

Formal supports such as primary health practitioners and psychologists, recommended at the indicated level for people showing signs of prolonged grief disorder, were used by approximately one in five participants. The levels of psychologist use in this study are higher than reported pre-pandemic [1], suggesting a higher level of distress during the pandemic, or greater awareness and acceptance of seeking help. People perceived formal support as helpful due to the objectivity they provided; however, in some cases they were perceived as unhelpful due to limited specialist skills in grief counselling and interventions. Several studies have shown that such knowledge is not included or well-integrated in the preparation of health professionals [40,41] and that concerns about the training and skill of providers is a reason why people hold negative attitudes about grief counselling [42]. Strategies to increase workforce capacity to support the increasing number of people bereaved now and for future pandemics are urgently needed. There are however multiple challenges facing the field in providing care under normal circumstances and gaps in the literature regarding treatment for prolonged grief. Whilst we may be able to identify prolonged grief earlier than 12 months and identify candidates of preventative care [43], it is less clear what effective early care for prolonged grief looks like. Preliminary work investigating an internet-based therapist-assisted prevention intervention for prolonged grief disorder has shown promising results [44], however there remains a scarcity of research in this area. In addition to different needs at different levels, people will also have different needs and different times. This is an area to be explored in analyses of longitudinal data from this study.

Concerningly, during the COVID-19 pandemic, twice as many people had unmet bereavement support needs compared to pre-pandemic reports [1,4]. Needs ranged from practical to emotional support and emerged before the death, at the time of death, and after the death. This highlights the complexity of supporting the bereaved and the need to develop a comprehensive action plan in collaboration with a wide range of stakeholders. These include people involved in the care of the dying (where relevant), and people involved in care of the family after the death. In other words, work is needed to actualise a continuum of bereavement support that begins with end-of-life care [45]. Additionally, a key area of unmet need specific to the pandemic context was clarity of communication from government, clinicians, and public health announcements about restrictions. People reported confusion about what the restrictions were at a given time and sought clarification for compassionate visits and exemptions without success which added stress. The rationale for public health protections and clarity and simplicity of processes for seeking and being granted exemptions must be addressed in planning for future pandemics.

The strongest correlates of unmet support needs were experiencing the death of a partner or child. A close relationship to the deceased was also a significant correlate of poorer mental health outcomes in the sample [30]. Inability to spend time with the dying person, being unaware of what was happening to them, and being unable to say goodbye due to restrictions were also independent correlates of unmet needs in this study. Public health measures necessarily consider possible impacts of a pandemic at a population level, rather than the individual level, and set regulations, accordingly. In circumstances, such as the first 12 months of the global pandemic, when much was unknown about COVID-19 and its mutations (e.g., transmissibility, likelihood of severe disease/death across age groups, effectiveness of vaccination), the precautionary principle drove more restrictive settings. At a population level, this approach in Australia was successful by international comparisons [46]. For individuals, however, (in this case, the bereaved), there were significant negative consequences related to those settings. Those responding to future pandemics might specifically consider carers of people in the final stages of life, their families, and the recently bereaved for possible modifications to some of the restrictions. At a practical level, spaces could be redesigned to allow for safe access to enable togetherness. For example, the built environment of nursing homes (many communal facilities) as well as the care provision required (close contact) are among several factors that enhance risk of transmission in this setting. Evidence suggests that nursing homes that experienced larger COVID outbreaks were those accommodating a larger number of residents, with more shared rooms, and more likely to report non-compliance with regulations [47]. This supports the redesign of the built environment of care spaces as well as adapted operations (compliance with sanitary measures) in the short and long term to better prepare us for future outbreaks and pandemics and enable opportunities for togetherness that are safe [47].

Finally, the Productivity Commission’s Inquiry into Mental Health 2022 emphasised clinical services over the importance of family and social networks in providing support [48]. Given that social supports were relied on most, perceived as most helpful, and lack thereof independently accounted for variance in unmet support needs, a clear recommendation for a bereavement support action plan is to bolster social networks’ ability to provide support in times of loss; although must not be used as the only strategy. As suggested by the public health model of bereavement support, formal specialist grief therapy is required for some, but all levels of support are needed [1].

### Strengths and limitations

This is the largest Australian study to report on bereavement support during the COVID-19 pandemic. There are, however, some limitations and cautions to be taken in interpreting findings. Participants were mostly female and English speaking, and a convenience sample who were recruited online which limits representativeness. There may be gender differences in bereavement support needs [49] that were not captured in this sample. Recruitment via social media allowed for capture of a large sample across Australian states as over 90% of Australians report smartphone, internet and social media use [50], however, people with limited internet access and or limited digital literacy may be underrepresented. Additionally, the online questionnaire reported here captured data at a single point in time and thus we are not able to infer causality from the correlations. Also, the list of supports in the questionnaire, while developed from previous literature was not exhaustive. There might have been other supports people used and found helpful; however, participants could indicate as such in the ‘other’ option. No other salient support sources emerged. We were also focused on people’s experience with external forms of supports, however, and did not index potential self-regulation strategies such as exercise or meditation.

## Conclusions

This study demonstrated the complexity of bereavement support needs during a pandemic. Specialised grief therapy needs to be more readily available to the minority of grievers who would benefit from it. In tandem, because two-thirds of participants reported unmet support needs, the urgency and necessity of bolstering bereavement support at every level—universal, selective, indicated—is highlighted. Support in the wake of bereavement is essential to help the increasing number of people bereaved now and for future pandemics.

## Supporting information

S1 File Questions capturing COVID-19 impacts. (DOCX)

S2 File Recruitment flow diagram. (DOCX)

S3 File The experience of COVID-19 restrictions by support needs. (DOCX)

S4 File Ten most frequently reported helpful and unhelpful supports during COVID-19. (DOCX)
