# Supplementary figures and images for: Support needs of Australians bereaved during the COVID-19 pandemic: A cross-sectional survey study
Source: PLoS One. 2024 Jun 6;19(6):e0304025. doi: 10.1371/journal.pone.0304025 (PMC11156310; doi:10.1371/journal.pone.0304025)

## Additional file 1 – survey questions capturing COVID-19 impacts


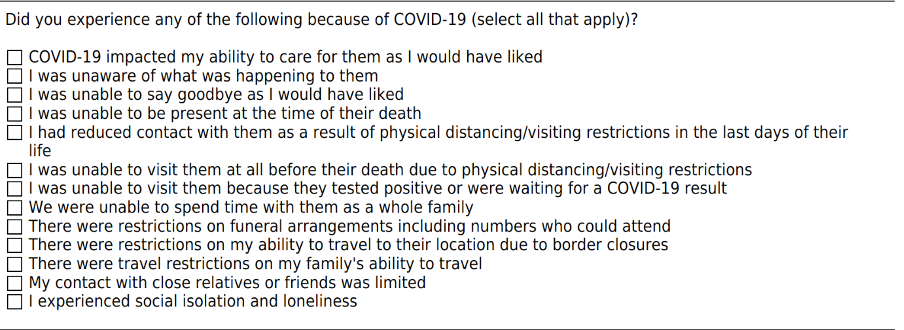

Supplement: S1 File — (DOCX) [file pone.0304025.s001.docx]

## Additional file 2 – recruitment flow diagram


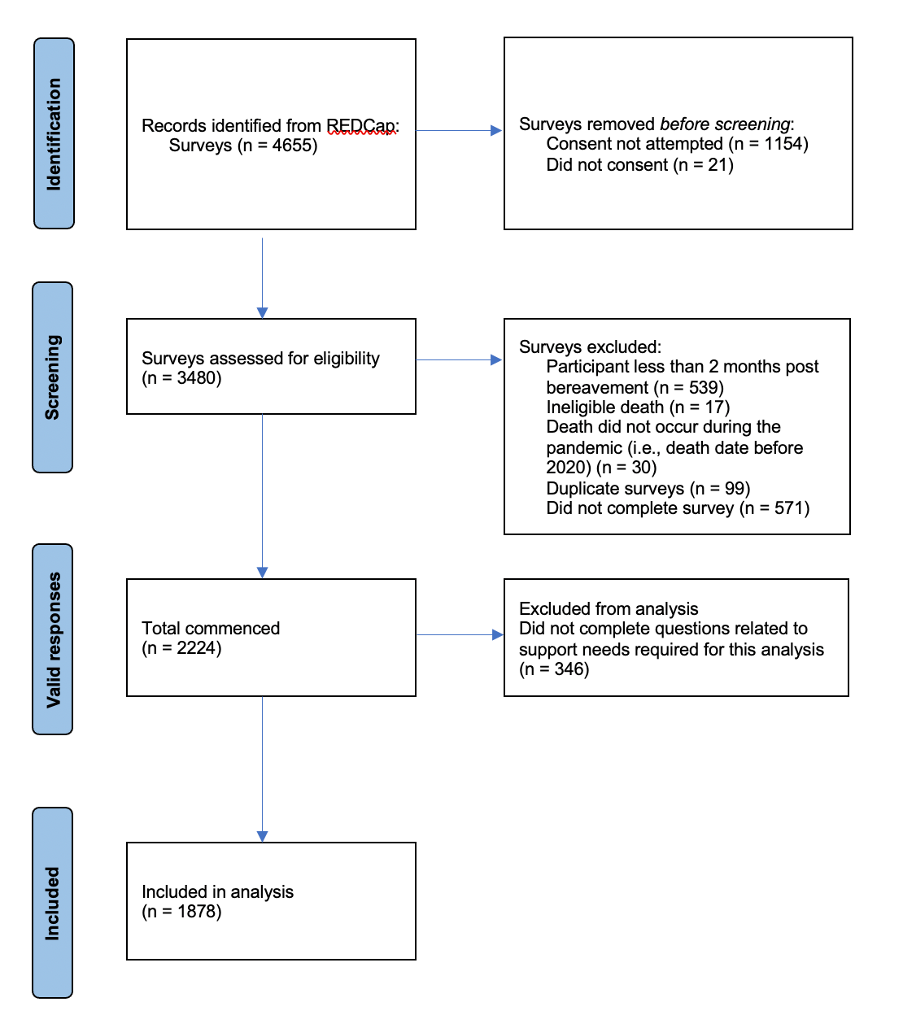

Supplement: S2 File — (DOCX) [file pone.0304025.s002.docx]
