# Supplementary material for: Support needs of Australians bereaved during the COVID-19 pandemic: A cross-sectional survey study
Source: PLoS One. 2024 Jun 6;19(6):e0304025. doi: 10.1371/journal.pone.0304025 (PMC11156310; doi:10.1371/journal.pone.0304025)
Supplement: S3 File — (DOCX) [file pone.0304025.s003.docx]

## Additional file 3 – the experience of COVID-19 restrictions by support needs

|  | Total  (*n*=1,878) | Support needs met  (*n*=714) | Unmet support needs  (*n*=1,164) | *p*-value |
| --- | --- | --- | --- | --- |
|  | ***n* (%)** |  |  |  |
| *The experience of COVID-19 restrictions* |  |  |  |  |
| Before the death |  |  |  |  |
| We were unable to spend time with them as a whole family | 925 (49.3) | 309 (43.3) | 616 (52.9) | <.001 |
| There were travel restrictions on my family’s ability to travel | 888 (47.3) | 313 (43.8) | 575 (49.4) | .020 |
| COVID-19 impacted my ability to care for them as I would have liked | 858 (45.7) | 253 (35.4) | 605 (52) | <.001 |
| I was unable to visit them at all before their death due to physical distancing/visiting restrictions | 534 (28.4) | 187 (26.2) | 347 (29.8) | .092 |
| There were restrictions on my ability to travel to their location due to border closures | 461 (24.5) | 179 (25.1) | 282 (24.2) | .699 |
| I was unaware of what was happening to them | 350 (18.6) | 86 (12) | 264 (22.7) | <.001 |
| I was unable to visit them because they tested positive or were waiting for a COVID-19 result | 39 (2.1) | 11 (1.5) | 28 (2.4) | .244 |
| At the time of death |  |  |  |  |
| I was unable to say goodbye as I would have liked | 851 (45.3) | 282 (39.5) | 569 (48.9) | <.001 |
| I had reduced contact with them as a result of physical distancing/visiting restrictions in the last days of their life | 847 (45.1) | 285 (39.9) | 562 (48.3) | <.001 |
| I was unable to be present at the time of their death | 663 (35.3) | 230 (32.2) | 433 (37.2) | .029 |
| After the death |  |  |  |  |
| There were restrictions on funeral arrangements including numbers who could attend | 1314 (70) | 473 (66.2) | 841 (72.3) | .007 |
| My contact with close relatives or friends was limited | 979 (52.1) | 288 (40.3) | 691 (59.4) | <.001 |
| I experienced social isolation and loneliness | 897 (47.8) | 182 (25.5) | 715 (61.4) | <.001 |
