# Supplementary material for: Support needs of Australians bereaved during the COVID-19 pandemic: A cross-sectional survey study
Source: PLoS One. 2024 Jun 6;19(6):e0304025. doi: 10.1371/journal.pone.0304025 (PMC11156310; doi:10.1371/journal.pone.0304025)
Supplement: S4 File — (DOCX) [file pone.0304025.s004.docx]

## Additional file 4 – ten most frequently reported helpful and unhelpful supports during COVID-19

| 10 most frequently reported helpful supports (n=1878) | n (%)^d^ | 10 most frequently reported unhelpful supports (n=1429) | n (%)^d^ |
| --- | --- | --- | --- |
| Family and friends | 971 (66.7) | None were unhelpful | 398 (27.9) |
| Psychologist | 130 (8.9) | Family and friends | 341 (23.9) |
| Self-help resources^a^ | 71 (4.9) | Government and lockdown | 87 (6.1) |
| Grief counseling | 71 (4.9) | Psychologist | 67 (4.7) |
| Internet/online community support groups^b^ | 43 (2.9) | General practitioner | 51 (3.6) |
| General practitioner | 33 (2.3) | Legal/financial support | 34 (2.4) |
| Other^c^ | 27 (1.9) | Grief counselling | 32 (2.2) |
| Religious leaders/organisations | 22 (1.5) | Aged care | 30 (2.1) |
| Palliative care service | 10 (0.7) | Palliative care | 28 (2.0) |
| Psychiatrist | 10 (0.7) | Self-help resources | 24 (1.7) |

^a^ e.g. information on grief related websites, books

^b^ e.g. Facebook

^c^ e.g. Meditation, hypnotherapist, alcohol

^d^ Total % does not add up to 100% as only the top ten are reported in this table
